# Minimally invasive cervical spine stabilization: A multicenter study of completely percutaneous pedicle screw-rod instrumentation

**DOI:** 10.1016/j.bas.2026.105996

**Published:** 2026-02-26

**Authors:** Jan-Helge Klingler, Lars Wessels, Christoph Scholz, Tobias Philip Schmidt, Ulf Bertram, Marc Hohenhaus, Ulrich Hubbe, Claudius Jelgersma, Nils Hecht, Ralf Watzlawick, Florian Volz, Jürgen Beck, Julia Onken, Ralph Kothe, Peter Vajkoczy, Hans Clusmann, Christian Blume

**Affiliations:** a Department of Neurosurgery, Medical Center - University of Freiburg, Faculty of Medicine, University of Freiburg, Germany; b Spine Science Commission of the German Spine Society (DWG), Hamburg, Germany; c Department of Neurosurgery, Charité - Universitätsmedizin Berlin, Berlin, Germany; d Department of Neurosurgery, RWTH Aachen University, Aachen, Germany; e Spine Department Schön Klinik Hamburg-Eilbek, Hamburg, Germany

**Keywords:** Cervical spine, Minimally invasive, Navigation, Pedicle screws, Percutaneous, Stabilization

## Abstract

**Introduction:**

Minimally invasive posterior cervical fixation remains technically challenging due to narrow pedicle dimensions and proximity to neurovascular structures. This multicenter study evaluates the accuracy and safety of a dedicated minimally invasive cervical pedicle screw-rod system with 3D navigation guidance.

**Research question:**

Can minimally invasive cervical pedicle screw placement achieve accuracy rates comparable to open techniques while maintaining patient safety?

**Material and methods:**

Retrospective multicenter analysis of 46 patients (60.7 ± 17.4 years) undergoing percutaneous cervical pedicle screw-rod instrumentation at three German university centers (01/2022–04/2024). Indications included degenerative disease (n = 21), tumors (n = 13), trauma (n = 8), and inflammation (n = 4). Primary outcome was neurological status (Frankel classification); secondary outcomes included screw accuracy (Bredow classification), surgical characteristics, and complications. All procedures utilized 3D navigation based on cone-beam CT or intraoperative CT.

**Results:**

In total, 232 pedicle screws were implanted from C2 to T2. Favorable screw position (Bredow grades 1–2) was achieved in 89.7% overall, with significantly lower accuracy at C3–C6 versus other levels (86.0% vs. 95.4%, p = 0.0297). No permanent neurological deficits occurred. Two screws required intraoperative repositioning; zero revision surgeries were needed. Mean surgical duration was 148 ± 66min with blood loss of 236 ± 183 ml.

**Discussion and conclusion:**

Minimally invasive cervical pedicle screw-rod instrumentation with 3D navigation achieves high accuracy and safety comparable to open techniques. In the majority of cases, the technique supplemented anterior fusion for additional stability, but it may also serve as posterior-only instrumentation for in tumor-related osteolysis or traumatic injuries. Mid-cervical levels remain particularly challenging and require heightened vigilance.

## Highlights

• First multicenter study on fully percutaneous cervical pedicle screw-rod fixation.
• 89.7% favorable screw accuracy with 3D navigation across three specialized centers.
• Zero permanent neurological deficits and zero wound healing complications observed.
• Improved accuracy in the second half of the study period suggests a learning curve.
• Mid-cervical levels (C3-C6) showed lower accuracy requiring heightened attention.

## Graphical abstract

**Figure:**
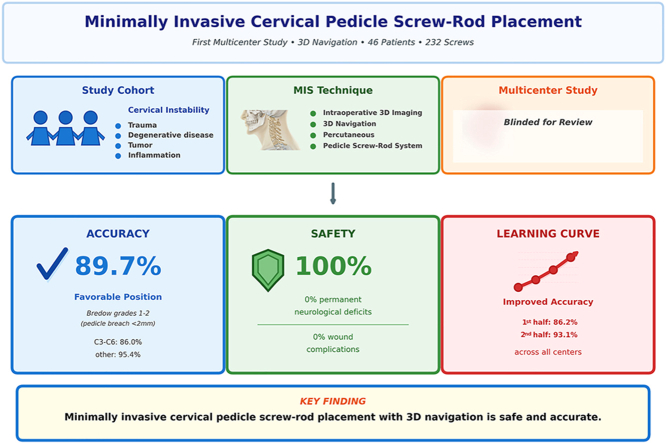

## 1 Introduction

Posterior screw-rod instrumentation is a well-established procedure for treating cervical instability caused by trauma, degenerative disease, tumors, or inflammation. Traditionally, posterior stabilization of the cervical spine required open exposure of large portions of the affected levels in order to insert screws and rods. In the thoracic and lumbar spine, minimally invasive surgical (MIS) techniques have shown advantages over conventional open surgical techniques, such as reduction of wound infections (O'Toole et al., 2009; Zhou et al., 2020; McGirt et al., 2011), intraoperative blood loss (Khan et al., 2015; Kim et al., 2020; Pokorny et al., 2022; Alshareef et al., 2021), postoperative pain (Khan et al., 2015), analgesic requirements (Kim et al., 2020; Wang et al., 2017), and postoperative length of stay (Khan et al., 2015; Kim et al., 2020; Pokorny et al., 2022). For the cervical spine, a cadaver study (Schmeiser et al., 2025) and preliminary monocentric clinical studies (Scholz et al., 2025; Blume et al., 2025; Jelgersma et al., 2026) have provided initial insights into this new MIS procedure involving the complete implantation of a dedicated and commercially available MIS screw-rod system. These previous studies found favorable MIS pedicle screw placement (pedicle breach <2 mm) in 84.4–90.0% (Schmeiser et al., 2025; Scholz et al., 2025; Blume et al., 2025; Jelgersma et al., 2026) of all inserted screws.

The present multicenter study comprises an extended retrospective dataset from the three monocentric clinical studies (Scholz et al., 2025; Blume et al., 2025; Jelgersma et al., 2026). This makes it the largest compilation of data to date on completely MIS cervical pedicle screw-rod instrumentation, focusing on short-term clinical outcomes and pedicle screw accuracy.

## 2 Methods

### 2.1 Study population

This is a multicenter retrospective cohort study conducted at three German neurosurgical university centers (Aachen, Berlin, and Freiburg). The study included consecutive adult patients with cervical instability caused by trauma, tumor, inflammation, or degenerative disease, who underwent implantation of a MIS cervical pedicle screw-rod system (Ennovate Cervical MIS, Aesculap, B. Braun, Melsungen, Germany) from January 2022 to April 2024 (Schmeiser et al., 2025; Scholz et al., 2025; Blume et al., 2025).

The lead ethics committee approved the study (reference number 24-1059_1-S1-retro). No formal patient consent was required for this type of retrospective study. The study was registered in the German Clinical Trials Register (DRKS00033215).

### 2.2 Outcome measures

The primary outcome was postoperative neurological status according to the Frankel classification (Frankel et al., 1969) compared with preoperative baseline. Secondary outcomes included screw accuracy on postoperative computed tomography (CT) according to the Bredow classification (Bredow et al., 2016), pedicle dimensions (convergence angle, height, and width) on preoperative CT, surgical characteristics, perioperative complications, and revision surgeries. The Bredow classification comprises five grades: Grade 1 indicates a screw position completely within the pedicle or a pedicle wall breach <1 mm, Grade 2 < 2 mm, Grade 3 < 3 mm, and Grade 4 < 4 mm. Grade 5 is defined as a breach >4 mm and/or encroachment on the transverse foramen by more than half of the screw diameter. While the Bredow classification does not systematically code for breach direction (medial, lateral, superior, inferior) - except for Grade 5 which specifically indicates lateral breach - our multicenter study applied this established classification for consistency, acknowledging that breach direction can involve varying clinical risks. Grades 1 and 2 were considered favorable screw positions (Scholz et al., 2025). The pedicle parameters were measured radiographically by two independent raters, and the mean values of both measurements were used for subsequent analyses.

### 2.3 Surgical technique

Patients were placed in the prone position under general anesthesia. The head was fixed in neutral alignment using a Mayfield clamp. After firmly attaching the navigation reference clamp to a cervical spinous process, an intraoperative 3D cone-beam CT (CBCT) scan (e.g., Ziehm Vision RFD 3D, Ziehm Imaging, Nuremberg, Germany; O-arm, Medtronic, Minneapolis, MN, USA; Loop-X, Brainlab, Munich, Germany) or an intraoperative CT (iCT; e.g., Airo TruCT, Stryker, Kalamazoo, MI, USA) scan was acquired and transferred to a navigation system (e.g., Stryker NAV3i, Stryker, Kalamazoo, MI, USA; Curve Navigation, Brainlab, Munich, Germany). The lateralized skin incisions were planned using 3D navigation. Using a transmuscular MIS approach and the navigated guide handle, transpedicular trajectories were prepared with a high-speed drill and secured with K-wires. Individually dimensioned percutaneous pedicle screws with long sleeves were inserted via the K-wires, and longitudinal rods were introduced in a minimally invasive manner and secured with nuts to the recommended torque. The navigation reference clamp and the sleeves of the pedicle screws were removed, and the wounds were closed in standard fashion (Schmeiser et al., 2025; Scholz et al., 2025; Blume et al., 2025).

### 2.4 Statistics

Statistical analyses were performed using IBM SPSS Statistics version 29 (IBM Corp., Armonk, NY, USA). Continuous variables are presented as mean ± standard deviation. Categorical variables were compared using Fisher's exact test. The association between pedicle parameters and screw accuracy was assessed using Pearson's correlation coefficient. Inter-rater reliability of measured pedicle parameters between two raters was evaluated using the intraclass correlation coefficient (ICC) based on a two-way mixed-effects model for absolute agreement, using single measurements and multiple raters (Hallgren, 2012; Koo and Li, 2016). ICC values were interpreted according to Koo et al. (Koo and Li, 2016) as poor (<0.50), moderate (0.50–0.75), good (0.75–0.90), or excellent (>0.90). A p-value <0.05 was considered statistically significant.

## 3 Results

### 3.1 Patient characteristics

This study includes 46 patients (41.3% female, age: 60.7 ± 17.4 years, BMI: 27.0 ± 6.6 kg/m^2^) treated with MIS posterior screw-rod fixation for cervical instability due to degeneration (21/46), tumor (13/46), trauma (8/46), or inflammation (4/46). In some cases, multiple levels were affected by the pathology. The distribution of all 87 levels affected by these pathologies was as follows: 2 at C2–C3, 8 at C3–C4, 22 at C4–C5, 25 at C5–C6, 20 at C6–C7, and 10 at C7–Th1.

### 3.2 Surgical characteristics

In 32/46 cases (69.6%), MIS posterior screw-rod fixation was preceded by an anterior procedure (23/32 anterior cervical discectomy and fusion (ACDF), 6/32 anterior cervical corpectomy and fusion (ACF), 3/32 combination of ACDF and ACF). In the majority of cases, MIS posterior screw-rod fixation was preceded by an anterior procedure (ACDF/ACF), suggesting a primarily adjunctive role. In remaining cases, posterior instrumentation served as a posterior-only approach, with the specific goals (permanent stabilization vs. transient support pending fracture healing) determined by the underlying pathology.

**Fig. 1:**
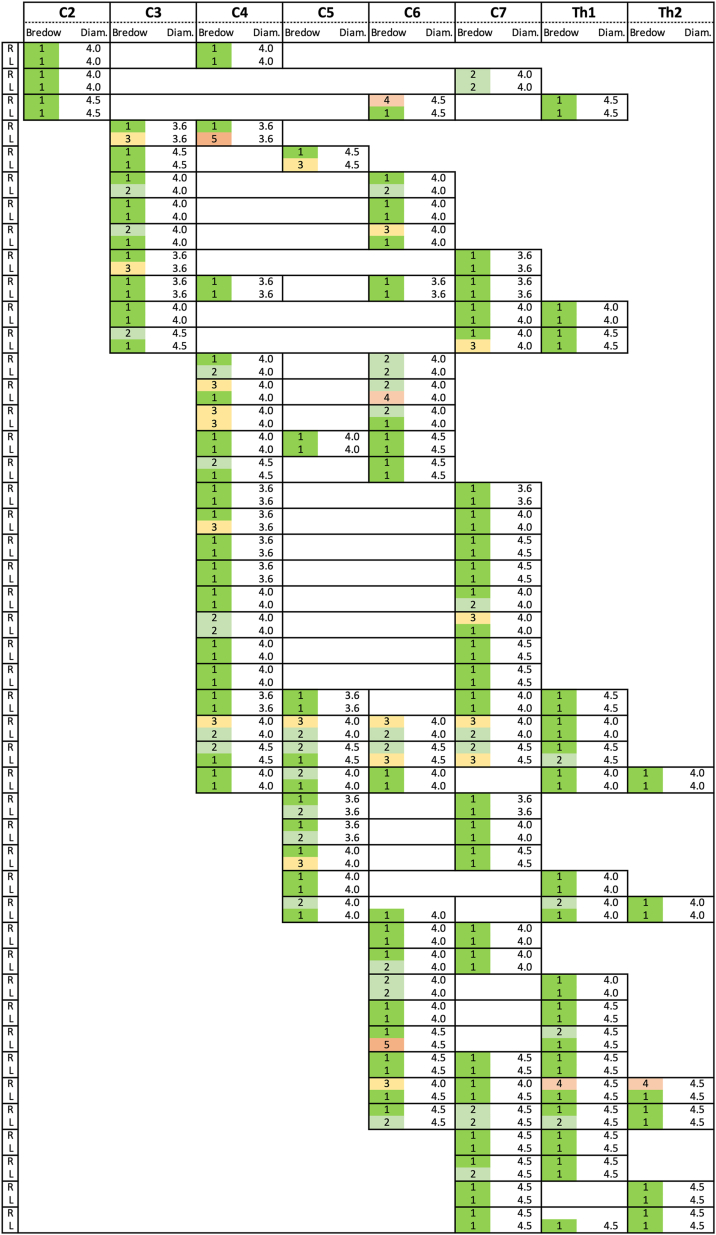
**Pedicle screw-rod distribution.** The figure shows the distribution of the 46 pedicle screw-rod implantations, the diameter of the implanted screws (Diam.), and screws placement accuracy evaluated using the Bredow classification on postoperative CT scans.

**Fig. 2:**
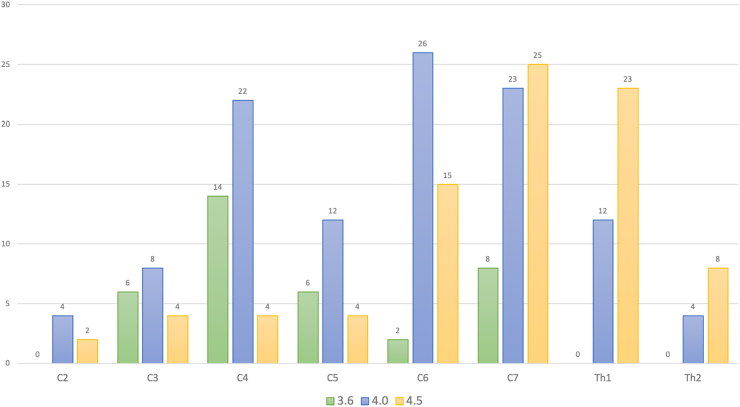
**Pedicle screw diameters.** The figure illustrates the frequency distribution of screw diameters (mm) across vertebral levels.

The distribution of pedicle screw-rod constructs and the 232 implanted pedicle screws is illustrated in Fig. 1, Fig. 2, respectively. Intraoperative 3D image acquisition was obtained using CBCT in 17 procedures and using iCT in 28 procedures (on case with missing documentation). The average number of intraoperative 3D scans was 2.3 ± 1.0. In most cases, the navigation reference clamp was attached to the spinous processes of C7 (38.1%) and C6 (23.8%). Concomitant MIS posterior decompression was performed in 23.9% of cases. Mean surgical duration for the posterior procedure (including any decompression) was 148 ± 66 min, and estimated blood loss was 236 ± 183 ml.

### 3.3 Neurological outcome

**Fig. 3:**
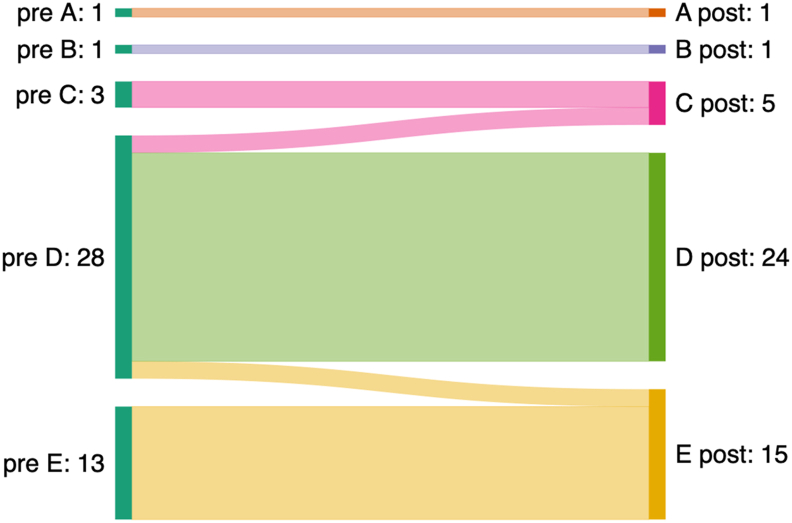
**Pre- and postoperative Frankel grading.** Pre- and postoperative neurological status according to the Frankel grading system. Most patients were graded Frankel D preoperatively; postoperatively, neurological status remained unchanged in 91.3% of patients; four patients changed by one grade (two deteriorated to grade C and two improved to grade E).

Preoperatively, the majority of patients had some functional impairment according to Frankel grade D (Fig. 3). Postoperatively, 42 of the 46 patients remained at their preoperative Frankel grade. In a total of four patients, the preoperative Frankel grade D changed by one grade: two of these patients deteriorated to grade C and two improved to grade E. Both patients with a worsening Frankel grade had favorable screw positions with no unusual findings during surgery. Both patients deteriorated during their longer-than-average hospital stay due to their underlying infectious disease.

Apart from this, one misplaced screw was repositioned intraoperatively without neurological sequelae. Another patient experienced transient coordination and gait impairment postoperatively after having a screw removed without replacement. A further patient developed a temporary mild C5 palsy with complete recovery after 6 weeks. In all other patients, no new postoperative deficits occurred. The mean postoperative length of stay was 10.5 ± 11.6 days.

### 3.4 Pedicle screw accuracy

**Fig. 4:**
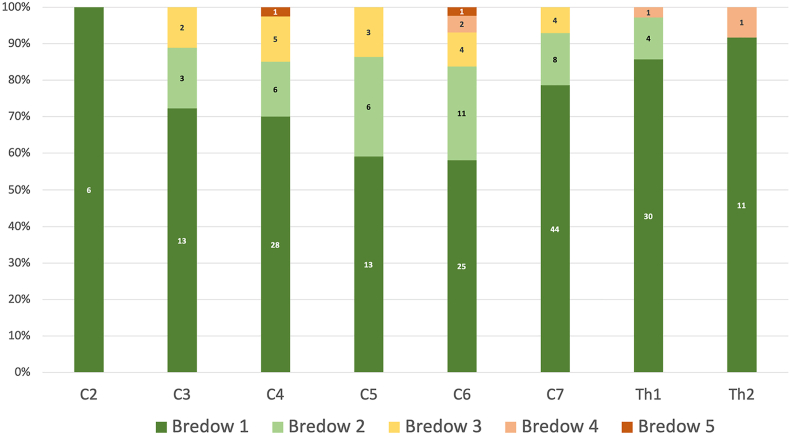
**Accuracy of percutaneous pedicle screws.** Accuracy of pedicle screw placement according to the Bredow classification. Favorable placement (Bredow grades 1–2) was achieved in 89.7% of screws; unfavorable grades were not associated with neurological deficits or revision. Favorable placement was less frequent at C3–C6 compared with other levels (86.0% vs. 95.4%, p = 0.0297).

A favorable position (Bredow grades 1 and 2) was achieved in 208 of 232 screws (89.7%) (Fig. 4). Two misplaced screws were removed intraoperatively as described above. Apart from this, screws graded as Bredow grade 3 (7.8%), Bredow grade 4 (1.7%), or Bredow grade 5 (0.9%) did not result in neurological deficits or radicular pain, and none required repositioning. Intraoperative conversion to an open screw insertion technique was not needed in any case. At C3–C6, favorable screw placement was achieved less frequently compared to the other vertebrae (86.0% vs. 95.4%, p = 0.0297) (Fig. 4).

**Fig. 5:**
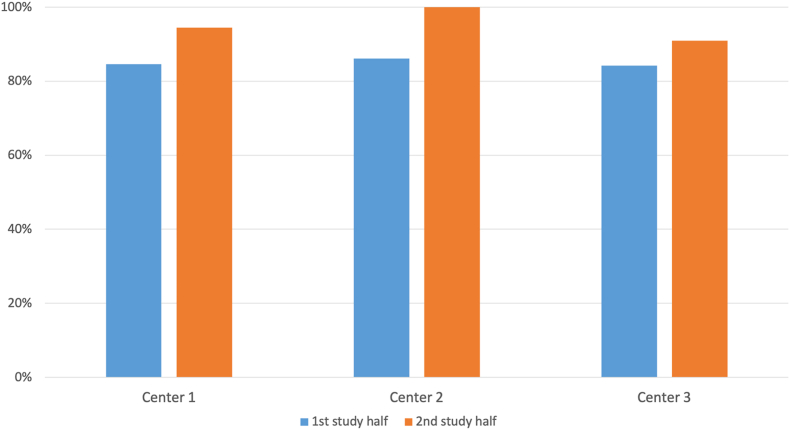
**Improved performance in the second half of the study.** Comparison of the first and second study halves showing a non-significant trend toward a higher proportion of favorable screw positions across all three centers in the second half.

In the second half of the study, a trend toward a higher proportion of favorable screw positions was observed across all three centers compared with the first half (Fig. 5).

### 3.5 Pedicle dimensions

We investigated the association between pedicle parameters and screw accuracy (favorable Bredow grades 1–2 versus unfavorable Bredow grades 3–5). No significant correlation was observed for any of the three parameters analyzed: pedicle width (r = −0.095, p = 0.149), pedicle height (r = −0.027, p = 0.679), and pedicle convergence angle (r = 0.116, p = 0.078).

The inter-rater reliability was “excellent” (>0.9) for the pedicle height (ICC 0.902; 95-CI: 0.875–0.923) and width (ICC 0.901; 95-CI: 0.872–0.923) and “good” for the measurement of the pedicle convergence angle (ICC 0.842; 95-CI: 0.800–0.876).

### 3.6 Medical complications

Medical complications not directly related to the surgical technique occurred in one patient who died of a pulmonary embolism on postoperative day 7 and in another patient who developed renal failure, sepsis, and endocarditis.

Wound healing complications did not occur, and revision surgery was not necessary in any case.

## 4 Discussion

This is the first multicenter study on completely MIS pedicle screw-rod fixation of the cervical spine. With 89.7% of screws achieving favorable positions, this retrospective study demonstrates high safety and precision of percutaneous pedicle screw placement in specialized spine centers with expertise in minimally invasive spine surgery as well as navigated pedicle screw placement. These results are comparable to – or exceed – previously reported accuracy rates for open pedicle screw placement techniques. The literature reports favorable screw positions of 74.4% for open pedicle screw placement at the cervicothoracic junction (Lee et al., 2007). A recent study found favorable screw positions in 67% of cases using conventional fluoroscopy and 88.6% using 3D navigation for open cervical pedicle screw placement (Bertram et al., 2021).

### 4.1 Clinical role and treatment strategy

The clinical role of posterior cervical pedicle screw-rod instrumentation must be understood within the context of specific pathologies and treatment goals. In this multicenter series, posterior instrumentation served distinct purposes depending on the underlying condition.

In the majority of cases, posterior instrumentation was performed as an adjunct following anterior fusion procedures (ACDF or ACF), providing supplemental biomechanical stability. This combined anterior-posterior approach is particularly indicated in multilevel corpectomy with substantial osteolytic destruction from metastatic disease, severe degenerative instability, or cases where anterior reconstruction alone may not provide sufficient immediate stability. Biomechanical studies have demonstrated that combined anterior-posterior instrumentation provides superior construct stability compared to anterior-only fixation. Finite element analysis shows that combined fixation shields both the graft-endplate interfaces and the screw-bone interfaces from peak stresses, providing the least amount of construct motion (Hussain et al., 2011). In multilevel cervical ACF, posterior segmental instrumentation confers significant stability to the fusion construct and should be strongly considered to improve overall stability after two-level ACF (Setzer et al., 2012).

In remaining cases, posterior instrumentation served as a posterior-only approach without concomitant anterior fusion. This approach was chosen when the underlying pathology predominantly involved posterior elements of the cervical spine, or when the traumatic injury pattern was considered sufficiently stabilizable with a posterior pedicle screw-rod construct alone. Moreover, this strategy allowed for the option of elective implant removal following successful healing of the traumatic lesion, potentially reducing long-term implant-related complications and preserving motion segments in appropriate patients.

The specific therapeutic goal varied according to pathology. In cases requiring permanent stabilization, metastatic involvement of the cervical spine frequently necessitates posterior fixation to provide mechanical stability and pain relief when the vertebral body has been compromised by tumor. Similarly, inflammatory spondylodiscitis with structural compromise and severe degenerative instability may require definitive posterior constructs to ensure reliable bone healing and provide sufficient support for the anterior column. In selected cases of traumatic cervical injuries in younger patients, particularly with isolated posterior column fractures, the potential for hardware removal following fracture consolidation may be considered to preserve segmental motion.

Decision-making considerations for treatment strategy consider the extent and distribution of pathology, the integrity of the anterior and middle spinal columns, patient age and functional requirements, and the need for decompression due to neurological impairment. The MIS pedicle screw-rod instrumentation offers versatility across these clinical scenarios with the potential benefits of the MIS technique.

### 4.2 Historical development and biomechanical superiority

Prior to screw fixation techniques, cervical spine stabilization relied on in-situ fusion or wiring techniques, using autologous bone grafts and postoperative external immobilization. Despite prolonged bracing, these approaches yielded considerable pseudoarthrosis rates (Farey et al., 1999; Joaquim et al., 2018). Since the 1970s, lateral mass screw fixation has become an established option for posterior cervical spine stabilization, with Roy-Camille credited for its first use in 1964 (Joaquim et al., 2018). However, dissatisfaction with lateral mass fixation, particularly at the cervicothoracic junction, led to the development of cervical pedicle screw insertion, as described by Abumi et al., in 1994 (Abumi et al., 1994) for traumatic cervical spine injuries.

Biomechanically, cervical pedicle screws demonstrated substantial superiority over lateral mass screws. Studies have shown significantly higher stability in lateral bending (Kothe et al., 2004), with pedicle screws yielding four times higher pullout strength after torsion loading and twice the pullout strength after flexion-extension loading (Ito et al., 2014; Johnston et al., 2006). This superior biomechanical stability permits shorter instrumentations with enhanced reduction capabilities, potentially eliminating the need for additional anterior procedures required with lateral mass screws (Tukkapuram et al., 2019).

### 4.3 Evolution of cervical pedicle screw placement techniques

Despite the biomechanical advantages of cervical pedicle screws, concerns regarding their insertion have historically centered on the potential risk of neurovascular complications due to the narrow pedicle width and the immediate proximity to critical structures, particularly the vertebral artery (Abumi et al., 1994; Kothe et al., 2004; Ito et al., 2014).

Soliman et al. (2023) compared subaxial cervical pedicle screws versus lateral mass screws in 4165 patients with 16,669 screws, demonstrating significantly fewer lateral mass fractures (odds ratio = 43.2), fewer revision surgeries (odds ratio = 5.6), and fewer infections (odds ratio = 5.5) with pedicle screws, supporting the safety profile observed in our study.

The accuracy of open cervical pedicle screw placement varies substantially depending on the insertion technique, surgical experience, and the accuracy definition used. Early freehand techniques without intraoperative image guidance achieved only 12.5–34.4% accuracy in cadaveric studies (Ludwig et al., 2000). The integration of conventional fluoroscopic guidance during open cervical pedicle screw insertion marked a decisive improvement, with experienced surgeons achieving accuracy rates up to 93% when defined as the absence of any pedicle breach (Abumi et al., 2000). The largest systematic review analyzing 14,118 cervical pedicle screws from 73 studies reported 85.3% accuracy for fluoroscopy-assisted and 84.4% for predominantly freehand techniques using this strict definition (Irmak et al., 2025), though with considerable variability in non-navigated techniques (0–43.5% breach rates) (Irmak et al., 2025) reflecting differences in surgical experience and learning curves (Yoshimoto et al., 2009).

Advanced 3D navigation techniques initially demonstrated superiority over conventional fluoroscopy. Early comparative studies using strict breach definitions reported 94–98.8% accuracy for navigation versus 88–93% for conventional fluoroscopy (Bertram et al., 2021; Kotani et al., 2003; Richter et al., 2005), with particularly pronounced benefits in degenerative cases (94% vs. 63%, p < 0.001) (Bertram et al., 2021). However, when using a clinically-oriented accuracy definition that accepts pedicle breaches up to 2 mm as acceptable, contemporary meta-analyses found no statistically significant differences between navigated (94%) and experienced fluoroscopy-assisted approaches (96%) (p = 0.582) (Bindels et al., 2024). Similarly, the largest systematic review found comparable overall accuracy between navigated (82.7%) and non-navigated techniques (85.3%) (Irmak et al., 2025), suggesting that experienced surgeons using optimized conventional fluoroscopic techniques can achieve accuracy comparable to 3D navigation-assisted methods. Overall complication rates remain low (1.3%), with vertebral artery injury in 0.21% of patients (Irmak et al., 2025), indicating that small pedicle breaches are often clinically well-tolerated.

### 4.4 Minimally invasive approach and clinical translation

Percutaneous cervical pedicle screw placement offers reduced soft tissue trauma, preservation of the posterior musculoligamentous complex, and improved biomechanical trajectories through muscle-sparing techniques (Coric et al., 2020). Traditional open approaches with extensive paraspinal muscle retraction can restrict optimal screw trajectories due to surrounding tissue tension (Shimokawa and Takami, 2017), a limitation circumvented by percutaneous techniques.

Coric and Rossi (2022) reported 27 patients undergoing percutaneous cervical pedicle screw-rod instrumentation (C1–C7) with 3D navigation, achieving two intraoperative revisions (7.4%) and a 3.7% reoperation rate without neurovascular injuries or infections.

### 4.5 Study findings in context

Our multicenter experience with MIS pedicle screw-rod fixation for cervical spine stabilization (46 patients, 232 screws) demonstrates reproducibility of this technique across specialized centers. The 89.7% favorable MIS pedicle screw position rate aligns with the findings of Tarawneh et al.'s (Tarawneh et al., 2021) systematic review on 4278 open cervical pedicle screws (17 studies, 1065 patients), which reported 3D navigation-assisted accuracy of 87.5% (range 79.5–97.5%). Our results are also in line with the recent findings of Brunken et al. (2025), who reported an accuracy of 89.3% and 89.7%, respectively, when comparing open versus percutaneous navigated pedicle screw placement for traumatic injuries of the subaxial cervical spine. Importantly, screws graded as Bredow grades 3–5 in our study (10.3%) did not result in neurological deficits or require repositioning. The learning curve observed across all centers, with improved accuracy in the second study half, reflects technical refinement and the learning process associated with 3D navigation systems.

### 4.6 Safety profile

3D navigation accuracy varies by vertebral level. Our study found significantly lower favorable placement rates at C3–C6 compared to other levels (86.0% vs. 95.4%, p = 0.0297). This finding directly corroborates the results reported by Shin et al. (2022), who reported mid-cervical (C3–C5) accuracy of 83.3–85% versus 93.3–100% for C2, C6, and C7. Greater C3–C5 mobility and vertebral rotation during instrumentation, together with small pedicle diameters, contribute to this consistent pattern across studies. Our analysis did not demonstrate a statistically significant association between pedicle dimensions and unfavorable screw placement.

No permanent neurological deficits occurred, comparing favorably to neurovascular complication rates reported for open cervical pedicle screw placement in recent systematic reviews (1.9% for fluoroscopy-guided and 0.3% for navigation-assisted techniques) (Tarawneh et al., 2021). Two patients experienced Frankel grade deterioration from their underlying infectious disease, not related to surgical complications; one temporary C5 palsy resolved completely. Zero wound healing complications and zero revision surgeries support the advantages of MIS techniques. Two screws required intraoperative revision; one of these revised screws was subsequently removed without permanent neurological consequences, apart from postoperative transient coordination and gait impairment, demonstrating navigation-guided safety margins. No revision surgery was necessary in any case.

### 4.7 Technical considerations

The average of 2.3 ± 1.0 intraoperative 3D scans balanced accuracy with radiation minimization. Mandelka et al. (2024) investigated cervical pedicle screw placement in 24 sawbone models using CBCT- and iCT-based navigation, reporting a 23-fold higher radiation dose with iCT. Reference clamp placement at C6 (23.8%) and C7 (38.1%) in our study aligns with recommendations for proximity to instrumented levels. MIS decompression was successfully integrated in 23.9% of cases without compromising accuracy.

### 4.8 Limitations

This retrospective study has inherent biases in patient selection and data collection. Although the multicenter design enhances generalizability, all participating centers were university hospitals with specific expertise in minimally invasive spine surgery, which may limit applicability to centers with less experience. The short follow-up period precludes assessment of long-term outcomes such as fusion rates or adjacent segment disease.

Although the Bredow classification provides validated assessment of screw accuracy, it does not systematically code breach direction except for Grade 5 (lateral breach with transverse foramen encroachment). The clinical relevance of breach direction - particularly regarding spinal canal and vertebral artery proximity - warrants future investigation with more granular directional classification systems.

Variation in imaging modalities (iCT in 28 cases, CBCT in 17 cases) may have influenced accuracy, though Mandelka et al. (2024) demonstrated comparable accuracy across modern navigation systems. No direct comparison group with open pedicle screw or lateral mass screw fixation was included. While operative time (148 ± 66 min) and blood loss (236 ± 183 ml) are acceptable, the minimally invasive advantage remains presumed without comparative data on postoperative pain, functional recovery, or patient-reported outcomes. To overcome these limitations and gain further insights into the potential benefits of this new technique, a multicenter randomized controlled trial was launched to compare minimally invasive and open cervical pedicle screw-rod instrumentation.

## 5 Conclusions

This multicenter study demonstrates that completely minimally invasive pedicle screw-rod fixation is a safe and accurate technique for cervical spine stabilization when performed by experienced surgeons with appropriate 3D navigation technology. The technique provides versatility to serve as an adjunct to anterior fusion procedures, as a posterior-only approach for permanent stabilization in tumor-related osteolysis or severe degenerative disease, or—in selected trauma cases—as transient support pending fracture healing. The 89.7% favorable screw position rate, zero wound healing complications, and zero permanent neurological deficits support this technique as a viable alternative to open approaches and lateral mass screws. The learning curve across centers emphasizes the importance of adequate training in minimally invasive spine surgery and navigation systems. Mid-cervical levels (C3-C6) require heightened vigilance given their consistently lower accuracy rates across published series. An ongoing randomized controlled trial will provide definitive comparative data on clinical outcomes relative to the conventional open technique.

## Previous presentations

Parts of this work were presented at the:

- Annual Meeting of the Spine Section of the German Society of Neurosurgery (DGNC) March, 28-29, 2025, Heidelberg, Germany,
- EUROSPINE Annual Meeting, October 22-24, 2025, Copenhagen, Denmark (abstract in *Brain and Spine*: https://www.sciencedirect.com/science/article/pii/S2772529425007040), and
- Annual Meeting of the German Spine Society (DWG) December 10-12, 2025, Wiesbaden, Germany (abstract in *Brain and Spine*: www.sciencedirect.com/science/article/pii/S2772529425014857).

## Disclosures

• JHK has received consulting fees, research funding, and lecture honoraria from B. Braun
• LW has received research funding, and lecture honoraria from B. Braun
• UH has received consulting fees and lecture honoraria from B. Braun
• RK has received consulting fees and lecture honoraria from B. Braun
• CB has received consulting fees, research funding, and lecture honoraria from B. Braun
• All other authors declare no conflicts of interest.

## Declaration of competing interest

The authors declare the following financial interests/personal relationships which may be considered as potential competing interests: Jan-Helge Klingler reports a relationship with B Braun SE that includes: consulting or advisory, funding grants, and speaking and lecture fees. Lars Wessels reports a relationship with B Braun SE that includes: funding grants and speaking and lecture fees. Ulrich Hubbe reports a relationship with B Braun SE that includes: consulting or advisory and speaking and lecture fees. Ralph Kothe reports a relationship with B Braun SE that includes: consulting or advisory and speaking and lecture fees. Christian Blume reports a relationship with B Braun SE that includes: consulting or advisory, funding grants, and speaking and lecture fees. If there are other authors, they declare that they have no known competing financial interests or personal relationships that could have appeared to influence the work reported in this paper.

## References

[bib1] Abumi K., Itoh H., Taneichi H. (1994). Transpedicular screw fixation for traumatic lesions of the middle and lower cervical spine: description of the techniques and preliminary report. J. Spinal Disord..

[bib2] Abumi K., Shono Y., Ito M. (2000). Complications of pedicle screw fixation in reconstructive surgery of the cervical spine. Spine.

[bib3] Alshareef M., Klapthor G., Alawieh A. (2021). Evaluation of open and minimally invasive spinal surgery for the treatment of thoracolumbar metastatic epidural spinal cord compression: a systematic review. Eur. Spine J..

[bib4] Bertram U., Schmidt T.P., Clusmann H. (2021). Intraoperative computed tomography-assisted spinal navigation in dorsal cervical instrumentation: a prospective study on accuracy regarding different pathologies and screw types. World Neurosurg..

[bib5] Bindels B.J.J., Dronkers B.E.G., Smits M.L.J. (2024). Accurate placement and revisions for cervical pedicle screws placed with or without navigation: a systematic review and meta-analysis. Glob. Spine J..

[bib6] Blume C., Schmidt T.P., Mueller C.-A. (2025). A new minimally invasive cervical pedicle screw (CPS) fixation system using intra-operative computed tomography-guided navigation. J. Spine Surg..

[bib7] Bredow J., Beyer F., Oppermann J. (2016). A novel classification of screw placement accuracy in the cervical spine. Technol. Health Care.

[bib8] Brunken F., Gierse J., Raisch P. (2025). Comparison of open and percutaneous navigated pedicle screw placement for traumatic injuries of the subaxial cervical spine. Brain Spine.

[bib9] Coric D., Rossi V. (2022). Percutaneous posterior cervical pedicle instrumentation (C1 to C7) with navigation guidance: early series of 27 cases. Glob. Spine J..

[bib10] Coric D., Rossi V.J., Peloza J. (2020). Percutaneous, navigated minimally invasive posterior cervical pedicle screw fixation. Int. J. Spine Surg..

[bib11] Farey I.D., Nadkarni S., Smith N. (1999). Modified gallie technique versus transarticular screw fixation in C1-C2 fusion. Clin. Orthop. Relat. Res..

[bib12] Frankel H.L., Hancock D.O., Hyslop G. (1969). The value of postural reduction in the initial management of closed injuries of the spine with paraplegia and tetraplegia. I. Paraplegia.

[bib13] Hallgren K.A. (2012). Computing inter-rater reliability for observational data: an overview and tutorial. TQMP.

[bib14] Hussain M., Nassr A., Natarajan R.N. (2011). Biomechanical effects of anterior, posterior, and combined anterior-posterior instrumentation techniques on the stability of a multilevel cervical corpectomy construct: a finite element model analysis. Spine J..

[bib15] Irmak Y., Peter F., Moser M. (2025). Accuracy and safety assessment of subaxial cervical pedicle screw instrumentation: a systematic review. Spine J..

[bib16] Ito Z., Higashino K., Kato S. (2014). Pedicle screws can be 4 times stronger than lateral mass screws for insertion in the midcervical spine: a biomechanical study on strength of fixation. J. Spinal Disord. Tech..

[bib17] Jelgersma C., Weber C.F., Früh A. (2026). Clinical applicability of percutaneous cervical pedicle screws: a retrospective matched-pair study. J. Neurosurg. Spine.

[bib18] Joaquim A.F., Mudo M.L., Tan L.A. (2018). Posterior subaxial cervical spine screw fixation: a review of techniques. Glob. Spine J..

[bib19] Johnston T.L., Karaikovic E.E., Lautenschlager E.P. (2006). Cervical pedicle screws vs. lateral mass screws: uniplanar fatigue analysis and residual pullout strengths. Spine J..

[bib21] Khan N.R., Clark A.J., Lee S.L. (2015). Surgical outcomes for minimally invasive vs open transforaminal lumbar interbody fusion: an updated systematic review and meta-analysis. Neurosurgery.

[bib22] Kim C.H., Easley K., Lee J.-S. (2020). Comparison of minimally invasive versus open transforaminal interbody lumbar fusion. Glob. Spine J..

[bib23] Koo T.K., Li M.Y. (2016). A guideline of selecting and reporting intraclass correlation coefficients for reliability research. J. Chiropract. Med..

[bib24] Kotani Y., Abumi K., Ito M. (2003). Improved accuracy of computer-assisted cervical pedicle screw insertion. J. Neurosurg..

[bib25] Kothe R., Rüther W., Schneider E. (2004). Biomechanical analysis of transpedicular screw fixation in the subaxial cervical spine. Spine.

[bib26] Lee G.Y.F., Massicotte E.M., Rampersaud Y.R. (2007). Clinical accuracy of cervicothoracic pedicle screw placement: a comparison of the “open” lamino-foraminotomy and computer-assisted techniques. J. Spinal Disord. Tech..

[bib27] Ludwig S.C., Kramer D.L., Balderston R.A. (2000). Placement of pedicle screws in the human cadaveric cervical spine: comparative accuracy of three techniques. Spine.

[bib28] Mandelka E., Wolf J., Medrow A. (2024). Comparison of different imaging devices and navigation systems for cervical pedicle screw placement: an experimental study on screw accuracy, screw placement time and radiation dose. Sci. Rep..

[bib29] McGirt M.J., Parker S.L., Lerner J. (2011). Comparative analysis of perioperative surgical site infection after minimally invasive versus open posterior/transforaminal lumbar interbody fusion: analysis of hospital billing and discharge data from 5170 patients. J. Neurosurg. Spine.

[bib31] O'Toole J.E., Eichholz K.M., Fessler R.G. (2009). Surgical site infection rates after minimally invasive spinal surgery. J. Neurosurg. Spine.

[bib32] Pokorny G., Amaral R., Marcelino F. (2022). Minimally invasive versus open surgery for degenerative lumbar pathologies:a systematic review and meta-analysis. Eur. Spine J..

[bib33] Richter M., Cakir B., Schmidt R. (2005). Cervical pedicle screws: conventional versus computer-assisted placement of cannulated screws. Spine.

[bib34] Schmeiser G., Blume C., Hecht N. (2025). Navigated percutaneous placement of cervical pedicle screws: an anatomical feasibility study. Brain Spine.

[bib35] Scholz C., Hohenhaus M., Hubbe U. (2025). First experience using a new minimally invasive screw-rod system for completely percutaneous pedicle screw fixation of the cervical spine. J. Neurol. Surg. Cent. Eur. Neurosurg..

[bib36] Setzer M., Eleraky M., Johnson W.M. (2012). Biomechanical comparison of anterior cervical spine instrumentation techniques with and without supplemental posterior fusion after different corpectomy and discectomy combinations: laboratory investigation. J. Neurosurg. Spine.

[bib37] Shimokawa N., Takami T. (2017). Surgical safety of cervical pedicle screw placement with computer navigation system. Neurosurg. Rev..

[bib38] Shin H.K., Jeon S.R., Roh S.W. (2022). Benefits and pitfalls of O-Arm navigation in cervical pedicle screw. World Neurosurg..

[bib39] Soliman M.A.R., Aguirre A.O., Khan S. (2023). Complications associated with subaxial placement of pedicle screws versus lateral mass screws in the cervical spine (C2-T1): systematic review and meta-analysis comprising 4,165 patients and 16,669 screws. Neurosurg. Rev..

[bib40] Tarawneh A.M., Haleem S., D'Aquino D. (2021). The comparative accuracy and safety of fluoroscopic and navigation-based techniques in cervical pedicle screw fixation: systematic review and meta-analysis. J. Neurosurg. Spine.

[bib42] Tukkapuram V.R., Kuniyoshi A., Ito M. (2019). A review of the historical evolution, biomechanical advantage, clinical applications, and safe insertion techniques of cervical pedicle screw fixation. Spine Surg Relat Res.

[bib43] Wang X., Borgman B., Vertuani S. (2017). A systematic literature review of time to return to work and narcotic use after lumbar spinal fusion using minimal invasive and open surgery techniques. BMC Health Serv. Res..

[bib44] Yoshimoto H., Sato S., Hyakumachi T. (2009). Clinical accuracy of cervical pedicle screw insertion using lateral fluoroscopy: a radiographic analysis of the learning curve. Eur. Spine J..

[bib45] Zhou J., Wang R., Huo X. (2020). Incidence of surgical site infection after spine surgery: a systematic review and meta-analysis. Spine.

